# Giant cell arteritis associated with intravenous zoledronic acid administration

**DOI:** 10.1093/jbmrpl/ziae015

**Published:** 2024-02-15

**Authors:** Meridith L Balbach, Jennifer R Hewlett, Robert A Wermers, Kenneth J Warrington, S Bobo Tanner, Erin Y Chew

**Affiliations:** Division of Rheumatology and Immunology, Vanderbilt University Medical Center, 1301 Medical Center Drive Nashville, Nashville, TN 37232, United States; Division of Endocrinology, Diabetes, Metabolism and Nutrition, Mayo Clinic, 200 First Street SW Rochester, MN 55905, United States; Division of Endocrinology, Diabetes, Metabolism and Nutrition, Mayo Clinic, 200 First Street SW Rochester, MN 55905, United States; Division of Rheumatology, Mayo Clinic, 200 First Street SW Rochester, MN 55905, United States; Division of Rheumatology and Immunology, Vanderbilt University Medical Center, 1301 Medical Center Drive Nashville, Nashville, TN 37232, United States; Division of Rheumatology and Immunology, Vanderbilt University Medical Center, 1301 Medical Center Drive Nashville, Nashville, TN 37232, United States

**Keywords:** osteoporosis, diseases and disorders of/related to bone, fracture prevention < practice/policy-related issues, antiresorptives < therapeutics

## Abstract

Bisphosphonates frequently provoke a cytokine-driven acute clinical response (ACR) characterized by fever, chills, arthralgias, and myalgias. More rarely, an association between aminobisphosphonates, such as alendronate and zoledronic acid, and rheumatologic and/or immune-mediated syndromes (RIMS) has been described. Herein we report 2 patients, one with a prior history of rheumatic disease and one without, who developed giant cell arteritis meeting the American College of Rheumatology 2022 criteria following zoledronic acid infusion. We subsequently review existing mechanistic and clinical literature supporting this link. The duration of symptoms and elevation of inflammatory markers may serve as indicators for differentiating between the more common ACR and less frequent but potentially morbid RIMS. Although the benefit of bisphosphonates will outweigh the risk of RIMS for most patients with high fracture risk, clinicians should be aware of this phenomenon to assist earlier diagnosis and treatment in affected individuals.

## Graphical Abstract

**Graphical Abstract:**
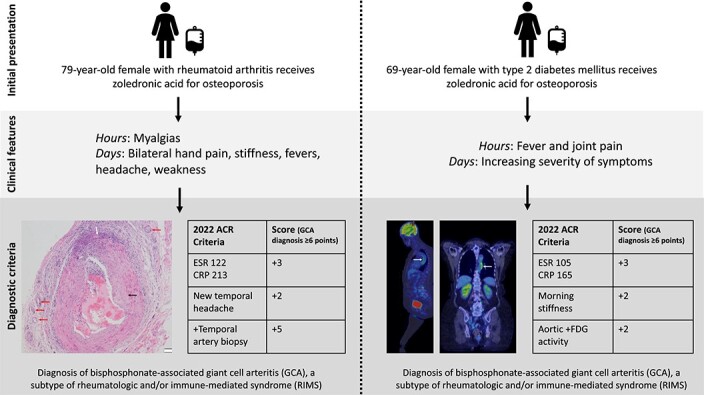

## Introduction

Bisphosphonates comprise a class of medications widely indicated in osteoporosis to reduce fractures. Aminobisphosphonates, such as alendronate and zoledronic acid, are nitrogen-containing pyrophosphate mimickers that inhibit farnesyl pyrophosphate at hydroxyapatite binding sites in bone tissue. ^1^ Inhibition of this key enzyme prevents attachment of osteoclasts to bone, thus disrupting bone resorption.

In addition to disruption of bone resorption, however, bisphosphonates are associated with upper gastrointestinal side effects, transient hypocalcemia, and more rarely, ocular adverse events (such as scleritis and uveitis), atypical femur fracture, and osteonecrosis of the jaw. Interestingly, an acute clinical response (ACR) characterized by fever, chills, arthralgias, and myalgias is observed in up to 42% of patients receiving their first dose of an intravenous (IV) aminobisphosphonate.^2^^,^^3^ Notably, those with an ACR with the first infusion appear to have a greater reduction in fracture risk despite no difference in the BMD response.^3^ This reaction may be explained by an indirect immunomodulatory effect of farnesyl pyrophosphate inhibition through accumulation of its substrate, isopentenyl pyrophosphate (IPP).^4^ IPP is an antigen that may be recognized by Vγ9δ2 T-cells independent of conventional MHC class presentation,^5^ resulting in T-cell activation and consequent release of proinflammatory cytokines such as elevated tumor necrosis factor alpha, IL-6, and interferon gamma.^6–8^ IPP-stimulated Vγ9δ2 T-cells may drive the ACR, as pamidronate was found to induce the activation and expansion of γδ T-cells, both ex vivo and in vivo among 4 (100%) patients experiencing an acute-phase reaction.^9^^,^^10^

Incidence of the cytokine-driven ACR invites speculation regarding the potential for bisphosphonates to amplify or induce inflammatory disorders. Indeed, there are prior reports of new-onset or exacerbated rheumatologic and/or immune-mediated syndromes (RIMS) associated with IV bisphosphonates.^11^^,^^12^ Because bisphosphonates are frequently indicated among patients with rheumatologic disease exposed to chronic steroid use, there is a need for better characterization of aminobisphosphonate-associated rheumatologic phenomena. Herein, we report a case series of 2 patients, one with a prior history of rheumatoid arthritis and another without known rheumatologic disease, who developed giant cell arteritis (GCA) associated with the use of zoledronic acid.

## Case 1 presentation

### Presentation

A 79-yr-old-woman with severe postmenopausal osteoporosis, rheumatoid arthritis, type 2 diabetes mellitus, and pyoderma gangrenosum was evaluated in the emergency department for severe body aches and subjective fever. She had been in her usual state of health until 6 d prior, when she developed myalgias a few hours after her initial dose of zoledronate IV 4 mg for osteoporosis. She subsequently developed bilateral hand pain and stiffness, causing difficulty with her activities of daily living such as using her cane and opening doors. Over the next several days, she developed progressively worsening subjective fevers, intermittent throbbing headache, generalized weakness, bilateral periorbital edema, and overall malaise, prompting her to seek medical attention. At presentation, she denied vision change or stiffness in her hips or shoulders. Prior to development of her symptoms, her rheumatoid arthritis had been stable on methotrexate 7.5 mg weekly and adalimumab 40 mg subcutaneous injection every 2 wk without a flare in over 10 yr.

On admission, she was febrile and hemodynamically stable. Examination revealed tenderness and effusion of the bilateral wrists and first and third metacarpophalangeal joints. Initial workup demonstrated normal baseline kidney function, normal white blood cell count, stable anemia, and elevated erythrocyte sedimentation rate (ESR) to 122 mm/h (normal, <37 mm/h) and C-reactive protein (CRP) to 213 mg/L (normal, <5 mg/L). She was evaluated by rheumatology, who noted the temporal association with zoledronate and diagnosed the patient with a rheumatoid arthritis flare. She was treated with a 10-d oral prednisone course.

Over the next several days, her pain and synovitis on exam improved, but her course was complicated by persistent fever, altered mental status, and new hypotension of unknown etiology requiring pressor support on hospital day 6. An empiric 7-d course of vancomycin and cefepime was started, and a broad infectious workup including blood cultures, urinalysis, hepatitis (A, B, and C) and tuberculosis screening, CT of the chest, abdomen, and pelvis, magnetic resonance angiography and venography of the head, transthoracic echocardiogram, and lumbar puncture was unrevealing. Her mental status and hypotension improved but she continued to be febrile and reported increasingly severe headache with unilateral temporal pain. Repeat ESR and CRP on hospital day 14 demonstrated persistently elevated ESR and CRP at 127 mm/h and 263 mg/L, respectively.

### Diagnosis

**Figure 1:**
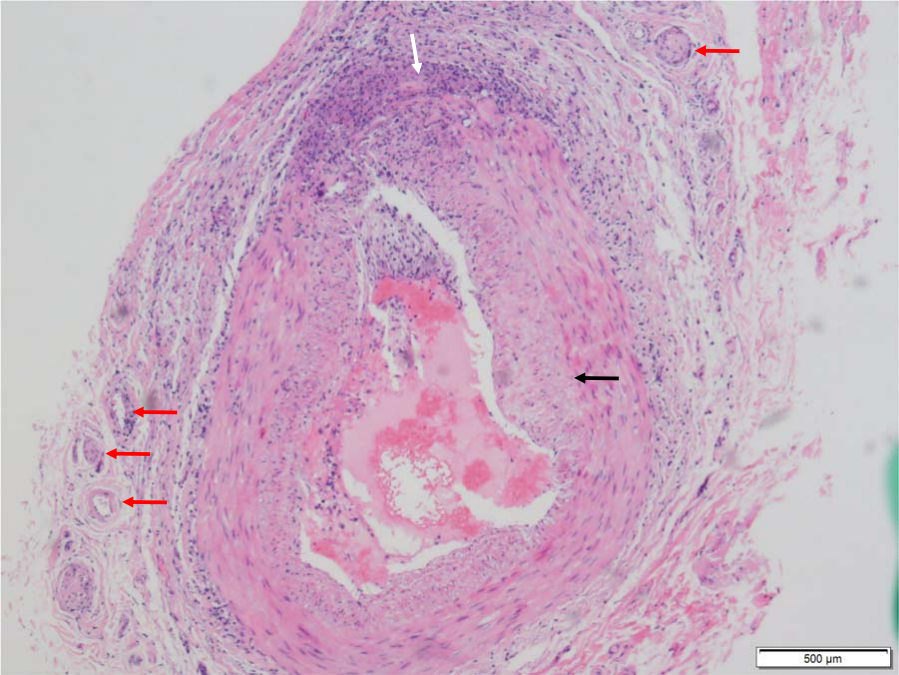
Medium size temporal artery biopsy with lymphohistiocytic inflammatory infiltrates between the media and adventitia (white arrow), intimal hyperplasia (black arrow), medial calcific sclerosis and associated focal elastin fiber fragmentation, and inflammation of vasa vasorum (red arrows). Numerous CD68+ histiocytes were present within the vessel wall. Based on clinical presentation, laboratory findings, and abnormal temporal artery biopsy, the patient met the 2022 American College of Rheumatology criteria for giant cell arteritis.^13^

The constellation of persistent fever, worsening intermittent headache with unilateral temporal pain, and elevated inflammatory markers was concerning for GCA. Bedside temporal artery ultrasound was negative for arterial wall edema (halo sign), and there was no contrasting echogenicity between the compressible arterial wall and surrounding tissue (compression sign). Right temporal artery biopsy was pursued and demonstrated lymphohistiocytic inflammatory infiltrates and arterial wall thickening, consistent with a diagnosis of active vasculitis (Figure 1).^13^

### Treatment

The patient was treated with prednisone 60 mg orally daily for 14 d with a plan to taper by 10 mg weekly and initiate tocilizumab IV 6 mg/kg monthly. Fevers and headaches resolved as inflammatory markers improved (ESR 102 mm/h and CRP 32 mg/L). One month later, she was asymptomatic with stable inflammatory markers (ESR 59 mm/h and CRP 57 mg/L).

## Case 2 presentation

### Presentation

A 69-yr-old woman with severe postmenopausal osteoporosis, type 2 diabetes, and Roux-en-Y gastric bypass presented for 10 d of subjective fever and arthralgias. Her medical history was notable for mildly elevated rheumatoid factor without a diagnosis of rheumatoid arthritis. She had developed a low-grade fever and small and large joint pain impacting her activities of daily living within 12 h of her initial infusion of zoledronic acid 5 mg for osteoporosis management. Severe symptoms persisted 10 d post-infusion, prompting further investigation. Initial findings were notable for a normal white blood cell count, negative blood cultures, and elevated CRP (165.8 mg/L, normal <5 mg/L). She was started on empiric doxycycline and methylprednisolone taper.

### Diagnosis

**Figure 2:**
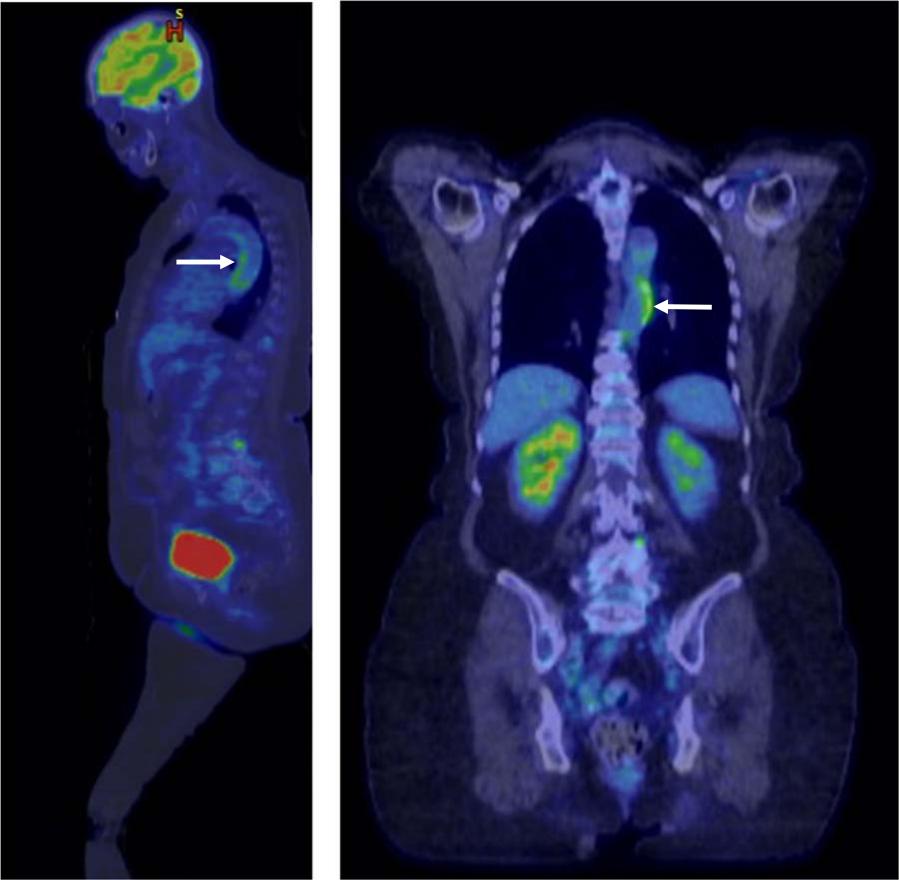
Fluorodeoxyglucose (FDG) PET-CT imaging with mild to moderate arterial FDG uptake most prominent in the descending aorta (white arrows). Based on clinical presentation, laboratory findings, and PET data, the patient met the 2022 American College of Rheumatology criteria for giant cell arteritis.^13^

She was referred to rheumatology, and clinical manifestations of inflammatory arthritis were confirmed. Initial findings were notable for an elevated ESR (105 mm/h, normal 2-22 mm/h) and a mildly elevated rheumatoid factor (31 IU/mL, normal <15 IU/mL). Further evaluation including antineutrophil cytoplasmic antibodies, antinuclear antibodies, cyclic citrullinated peptide antibodies, 14.3.3 ETA protein, anti-CEP-1 antibodies, and anti-Sa antibodies was negative. A cytokine panel revealed elevated TNF (34.0 pg/mL, normal <10.0 pg/mL), IL-6 (45.8 pg/mL, normal <5.0 pg/mL), interleukin-2 receptor (977 pg/mL, normal <959 pg/mL), and monocyte chemoattractant protein-1 (304 pg/mL, normal <198 pg/mL). Tuberculosis, hepatitis B, and hepatitis C screening were all negative. X-rays of the bilateral feet and hands showed degenerative changes in the first carpometacarpal joints and first metatarsophalangeal joints, all without erosions. Due to the marked elevation of inflammatory markers, additional imaging was obtained. Fluorodeoxyglucose (FDG) PET-CT revealed mild to moderate arterial FDG uptake in the descending aorta meeting classification criteria for GCA with a large vessel phenotype (Figure 2).^13^ Tissue diagnosis was not pursued, given the absence of cranial manifestations and previously reported sensitivity of temporal artery biopsy of 52% in GCA of a large vessel phenotype.^14^

### Treatment

She was started on prednisone 60 mg daily for large vessel GCA and seronegative inflammatory arthritis. Repeat CRP improved to 55 mg/L 2 wk later, and prednisone was tapered by 10 mg weekly thereafter. Two months later, the patient was asymptomatic on prednisone 10 mg daily with a CRP of 5.7 mg/L.

## Discussion

This case series describes 2 patients developing an ACR following aminobisphosphonate infusion that subsequently progressed to new-onset GCA, one with cranial disease and another with large vessel (aortic) involvement. Our findings agree with prior reports documenting similar occurrences of aminobisphosphonate-induced inflammatory disease (Table 1). A report by Gerster in 2004 of a 57-yr-old woman who developed polyarticular arthritis following oral alendronate use was followed by similar reports.^11^^,^^15–20^ Shared features include tender joints with effusion, often with elevated inflammatory markers and fever, similar to our patients. Although an ACR is less often seen in patients treated with oral bisphosphonates compared to IV bisphosphonates, inflammatory arthritis has been described in patients receiving both oral (*n* = 9)^11^^,^^15^^,^^19^ and IV bisphosphonates (*n* = 6).^12^^,^^16–18^ Some (*n* = 3) reported recurrence of symptoms with repeat aminobisphosphonate exposure.^15–17^ Although many of these cases represented a new inflammatory condition,^15^^,^^16^^,^^19^ some reported flare-up of previously documented osteoarthritis (*n* = 2)^17^^,^^18^ or a postoperative shoulder joint.^20^

**Table 1:** Summary of available literature describing rheumatologic and/or immune-mediated syndromes (RIMS).

Author	Sample size (*n*)^a^	Agent(s)	Inflammatory manifestation(s)	New-onset or preexisting	Supportive features^b^
Gerster^15^	1	Alendronate, oral 70 mg	Polyarticular arthritis	New-onset	5+ tender joints; arthrocentesis with WBC 6800 only; elevated ESR 16 mm/h & CRP 14 mg/L; recurrence with repeat exposure
Diaz-Borjon et al.^16^	1	Zoledronic acid IV 5 mg with pamidronate rechallenge IV 90 mg	Oligoarticular arthritis	New-onset	2 tender joints with effusion; recurrence with repeat exposure to same-class medication
Jones et al.^11^	7	Alendronate, oral 10 mg (*n* = 2), 70 mg (*n* = 5)	Mono (*n* = 2) or polyarticular (*n* = 5) arthritis	New-onset	Tender joint(s) with effusion; elevated ESR (*n* = 1), elevated CRP (*n* = 2) recurrence with repeat exposure in 5/5
Werner de Castro et al.^17^	1	Zoledronic acid IV 5 mg	Polyarticular arthritis	Preexisting osteoarthritis	5+ tender, erythematous, and warm joints; recurrence (though milder) with repeat exposure
White et al.^18^	1	Zoledronic acid IV 5 mg	Polyarticular arthritis	Preexisting osteoarthritis	5+ tender joints with effusion; fever; elevated CRP 163 mg/L
Gokkus et al. ^19^	1	Alendronate, oral 70 mg	Oligoarticular arthritis	New-onset	3 tender joints with effusion; elevated ESR 87 mm/h & CRP 50.8 mg/L
Miles et al.^20^	1	Zoledronic acid IV	Polyarticular arthralgia	New-onset in postoperative shoulder	Global arthralgias primarily affecting postoperative shoulder joint; resolution with oral steroids
Markovits et al.^12^	6	Zoledronic acid IV	Rheumatoid arthritis (*n* = 1), aromatase inhibitor-induced arthritis (*n* = 1), polymyalgia rheumatica (*n* = 2), Crohn’s disease (*n* = 1), autoimmune acquired hemophilia (*n* = 1)	New-onset (*n* = 5) and preexisting (*n* = 2)	Tender joints with effusion (*n* = 3); positive rheumatoid factor (*n* = 1); persistent musculoskeletal pain and elevated ESR and/or CRP (*n* = 2); autoimmune acquired hemophilia.
Metyas et al.^21^	1	Zoledronic acid IV	Giant cell arteritis	New-onset	Flu-like symptoms, left temporal headache and jaw pain, left eye pain and swelling, left temporal artery tenderness with diminished pulsation, complete left vision loss, elevated ESR 65 mm/h
Mahmood et al.^22^	81	Zoledronic acid or ibandronate	Giant cell arteritis	New-onset	Biopsy-proven giant cell arteritis within 90 d of infusion; unclear association
Marcu-Malina et al.^26^	1	Zoledronic acid IV	Digital thrombosis	Preexisting systemic sclerosis	Gangrene affecting fingers and toes

a For the purposes of this review, only patients with documented new-onset or exacerbated inflammatory condition temporally associated with aminobisphosphonate delivery are included in sample size.

b Erythrocyte sedimentation rate (ESR) and C-reactive protein (CRP) are listed, if available. Recurrence with re-exposure to aminobisphosphonate is noted, if applicable.

Interestingly, Markovits et al. identified 6 patients receiving zoledronic acid who had experienced new-onset or exacerbation of immune-mediated syndromes that included not only arthritis but also polymyalgia rheumatica (*n* = 2), Crohn’s disease flare (*n* = 1), progression of aromatase inhibitor-induced arthralgias to frank arthritis, and autoimmune acquired hemophilia.^12^ They found that this patient population experienced an ACR at higher rates versus those receiving zoledronic acid who do not develop a new-onset or exacerbation of inflammatory disease (83% vs 28%), linking this well-described cytokine-driven phenomenon to increasingly reported but rarer bisphosphonate-associated RIMS. Accordingly, both patients described herein demonstrated an ACR.

Notably, there has only been one well-documented case report of GCA after bisphosphonate infusion. Metyas et al. describe a patient who developed ACR after zoledronic acid with subsequent temporal headache, temporal artery tenderness, and monocular blindness, consistent with GCA. In contrast to this patient who had a nondiagnostic bilateral temporal artery biopsy, our patients had diagnostic histologic or PET-CT findings.^21^ More recently, Mahmood et al. published a retrospective cohort study based on Medicare claims data examining the incidence of GCA after zoledronic acid or ibandronate treatment.^22^ They compared their findings to previously reported nationwide incidence data and found no increase in the incidence of GCA after bisphosphonate infusion. However, the study was limited to Medicare patient data, a comparator cohort was not included, and the diagnosis of GCA was not verified by medical record review.

The mechanism by which bisphosphonates may induce GCA remains unknown. Study of cancer immune checkpoint inhibitor-mediated rheumatic disease, specifically GCA, may shed light on the pathophysiology driving bisphosphonate-associated RIMS. Administration of anti-PD-1 antibody into mouse models has been shown to increase infiltration of activated T-cells and IL-6 cytokines promoting hyperplasia in intima and adventitia.^23^ Analogously, IPP-stimulated Vγ9δ2 T-cell release of IL-6 may promote arterial wall inflammation.

Our work is limited by its retrospective nature, but this is expected given the rarity of this association. Although a relationship between aminobisphosphonate infusion and new-onset or exacerbated inflammatory disease is noted by several groups, causality cannot be established at present. Still, the aggregate work of our reports and that of others strongly supports the existence of an uncommon but well-defined incidence of RIMS temporally and mechanistically linked to aminobisphosphonate administration. Despite this, no such condition is described as a possible adverse reaction for any of the aminobisphosphonates. Increased awareness of this phenomenon is needed to enhance appropriate diagnosis and treatment.

The clinician may leverage the degree of inflammatory marker elevation to differentiate between bisphosphonate-associated RIMS over the more commonly seen ACR. The inflammatory response following IV zoledronate has been previously well-characterized by significantly increased granulocytes, IL-6, and CRP at 48 h as compared to baseline values measured prior to the infusion.^24^ Although the average CRP was elevated, it was much lower than that described in our cases, even among those with an ACR (49.4 mg/L ± 6.8 mg/L). There may be a role for CRP measurement when ACR symptoms last beyond 7 d, with investigation of underlying GCA if CRP is greater than 100 mg/L.

There are many remaining questions to be answered with potential implications for clinical practice. For example, what features predict who will develop a new-onset or exacerbated inflammatory disease with aminobisphosphonate infusion? There is some signal to suggest increased risk in those with prior malignancy or autoimmune disease, but better characterization is needed to counsel such individuals more accurately. Both of our patients had a history of diabetes. Interestingly, diabetes has been described as a protective risk factor for the ACR after zoledronic acid.^25^ Specific evaluation of patients with and without preexisting rheumatologic disease undergoing aminobisphosphonate infusion may help better estimate the incidence and characterize features of those most likely to develop bisphosphonate-associated RIMS. In most patients with high fracture risk, the benefit of treatment will outweigh the risk of RIMS, but improved awareness of this condition may assist in identifying individuals who were previously unrecognized.

## Data Availability

Additional non-identifiable data are available upon reasonable request of the corresponding author via email.
